# Correspondence

**Published:** 1864-02

**Authors:** 


[The following correspondence explains itself.—Ed. Jour.]

*Byfurorspeculoproulcerouterieumargentenitrate, &c.*, is the title of an essay which will teach young physicians how to make twelve thousand dollars per year. Mr. Editor, would you publish the same? If so, address

Dr. Cicatrix, P. O., Chicago.

---

Dear Dr. Cicatrix:

Yours of the —— *ult.* was duly received, but meanwhile—*Tempora mutantur et nos mutamur cum illis*—the Journal has passed into other hands. They permit, however, to reply to yours briefly. The publication of the essay is objected to:

1st. Because in these days of "shoddy" wealth is becoming a vulgarity, and its accumulation by a physician tends to impair the "dignity of the profession."

2nd. Because housebreaking, highway robbery and picking pockets, when successfully practiced, may yield the operator more than $12,000 a year, and hence the womb-burning practice would suffer by contrast.

3rd. Because it is not professional to show how "by indignities men come to dignities."

4th. Because there is danger of personalities, and the *impersonal* editorial "we" which "we" have just sloughed off, cannot abide the *personal;*—Speculo *ad alteram.*

5th. Because the ladder which takes one man to wealth and honor, oftentimes takes another to the pillory of popular contempt, or the richly merited hangman's noose.

6th. Because disclosures of unripe plans, or plans so ripe that they verge upon rottenness, of necessity, spoil the whole play, as the squeaking voice of "Bottom, the Weaver," dissipated the alarm of the audience when the leonine illusion waddled down to the footlights.

7th. Because carrying sandal-wood to the Chinese, now that everybody has learned the trick of it, is much like carrying coals to Newcastle.

8th. Because the imposture is fast unmasking itself.

9th. Because "we" have fifty other reasons, obvious to the cleanly mind, for not meddling with an emphatically "*dirty*" business, even though netting $12,000 a year. Ex-Ed.

